# Retropharyngeal Abscess With Severe Airway Compromise Following Anterior Cervical Spine Surgery: A Case Report

**DOI:** 10.7759/cureus.20754

**Published:** 2021-12-27

**Authors:** Brannon L Inman, Rachel E Bridwell, Neil P Larson, Sarah Goss, Joshua Oliver

**Affiliations:** 1 Emergency Medicine, Brooke Army Medical Center, Fort Sam Houston, USA; 2 Emergency Medicine, Madigan Army Medical Center, Tacoma, USA

**Keywords:** emergent airway, postoperative complicaiton, airway intubation, awake intubation, retropharyngeal mass, critical airway

## Abstract

Anterior cervical corpectomy, discectomy, and fusion are common surgical management options for symptomatic cervical radiculopathy and myelopathy. While these procedures are common and well-tolerated, postoperative complications span from mild dysphasia to airway compromise secondary to retropharyngeal or peri-cervical space abscess. These critical patients require robust airway management, which may entail a multidisciplinary approach or airway management in the operating room. We describe a patient who developed airway compromise 10 days following anterior cervical discectomy and fusion with a pre-platysmal abscess and a large retropharyngeal abscess. These abscesses were large enough to cause a mass effect with tracheal deviation. This deviation was severe enough that the patient required awake incision and drainage prior to rapid sequence intubation.

## Introduction

Cervical radiculopathy and myelopathy are typically treated via conservative measures such as physical therapy, though refractory cases may require anterior cervical corpectomy and fusion or anterior cervical discectomy and fusion (ACDF) for symptom control [1]. ACDF is a commonly performed and safe procedure, performed approximately 132,000 times annually with excellent outcomes and a low mortality rate of 0.1% [2]. The most common complaint is dysphagia, though up to 14% of patients experience some degree of airway discomfort [1,3]. Serious complications include postoperative edema, retropharyngeal hematoma, peri-cervical space abscess, and cerebrospinal fluid leakage [1]. We present a case of emergency airway compromise secondary to a retropharyngeal abscess (RPA) and pre-platysmal abscesses 10 days following multilevel ACDF.

## Case presentation

A 70-year-old female, status post ACDF of the 4th-7th cervical vertebrae, presented to the emergency department with two days of progressively worsening hoarse voice, sore throat, and dysphagia. She was evaluated by her neurosurgeon the day before presentation for this complaint and had been prescribed a commercially available methylprednisolone tapered dose pack. On presentation, her vital signs were as follows: blood pressure 159/76 mmHg, heart rate 93 beats per minute, respiratory rate 18 breaths per minute, temperature 36.9ºC (98.5ºF), and SpO_2_ 98%. Physical examination showed an ill-appearing woman with a hoarse voice, midline uvula, soft submandibular space, and pooling secretions controlled with self-suctioning. The patient maintained her torso slightly forward flexed, with the neck slightly extended. There was notable reluctance to changes in her position. Examination of the neck demonstrated an edematous fullness of the anterior neck with rightward tracheal deviation. The basic metabolic panel was within normal limits with the exception of hypokalemia at 3.1 mg/dL. Complete blood count demonstrated the following: hemoglobin of 13.6 g/dL, white blood cell count of 12.6 x 10^3^ cells/mm^3^, and platelets of 299 x 10^3^ cells/mm^3^. Erythrocyte sedimentation rate (ESR) and C-reactive protein (CRP) were 80 mm/hr and 21.7 mg/dL, respectively.

**Figure 1:**
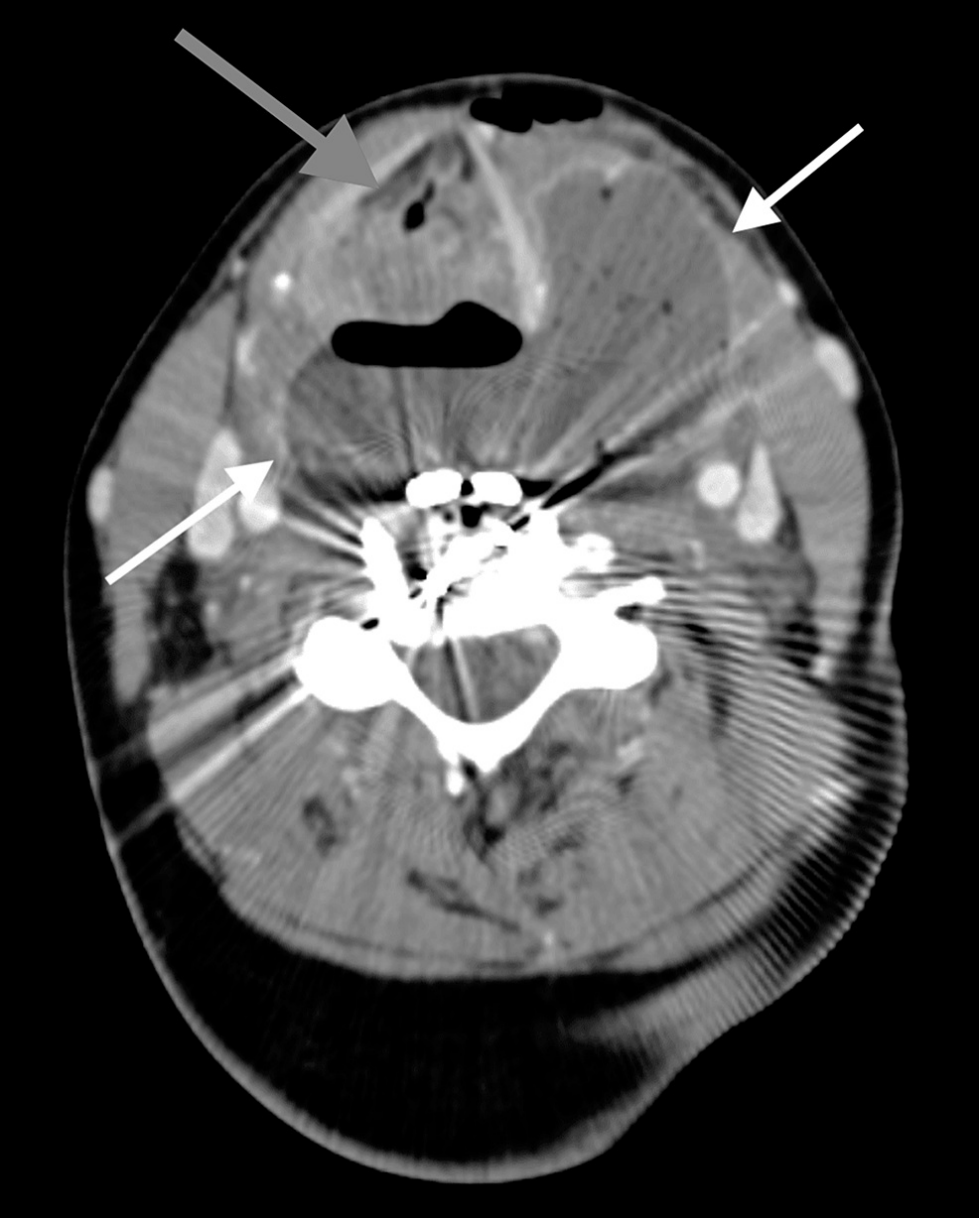
Transverse image of large retropharyngeal abscess with rightward mass effect on the trachea and severe glottic compression White Arrows: Large retropharyngeal abscess Grey Arrow: Tracheal displacement

**Figure 2:**
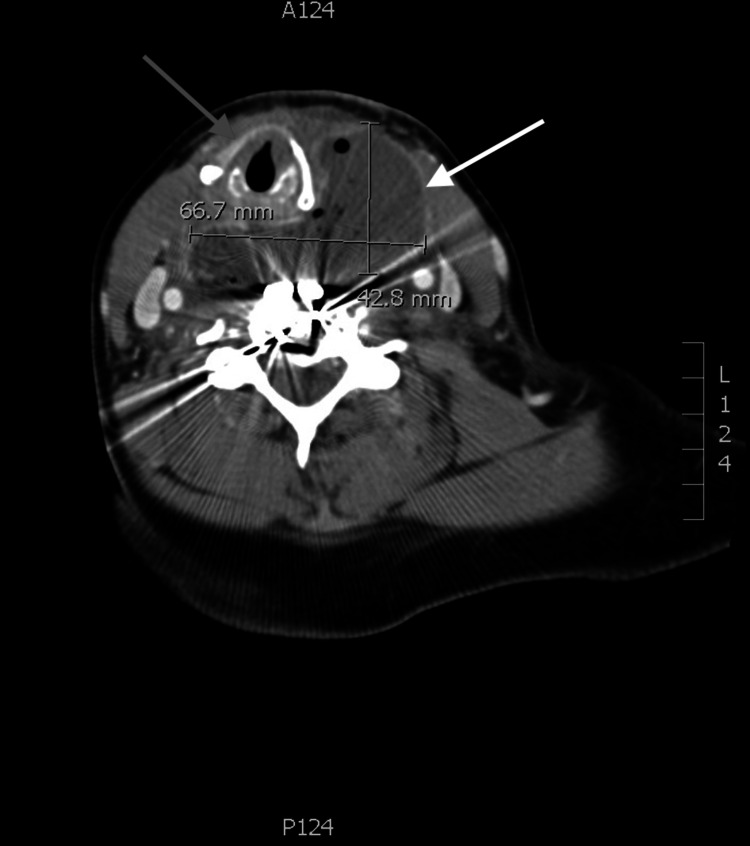
Transverse image of pre-platysmal abscess and a large retropharyngeal abscess measuring 6.67 cm x 4.28 cm in the transverse plane with rightward mass effect on the trachea White Arrow: Large retropharyngeal abscess measuring 6.67 cm x 4.28 cm Grey Arrow: Rightward mass effect on the trachea

**Figure 3:**
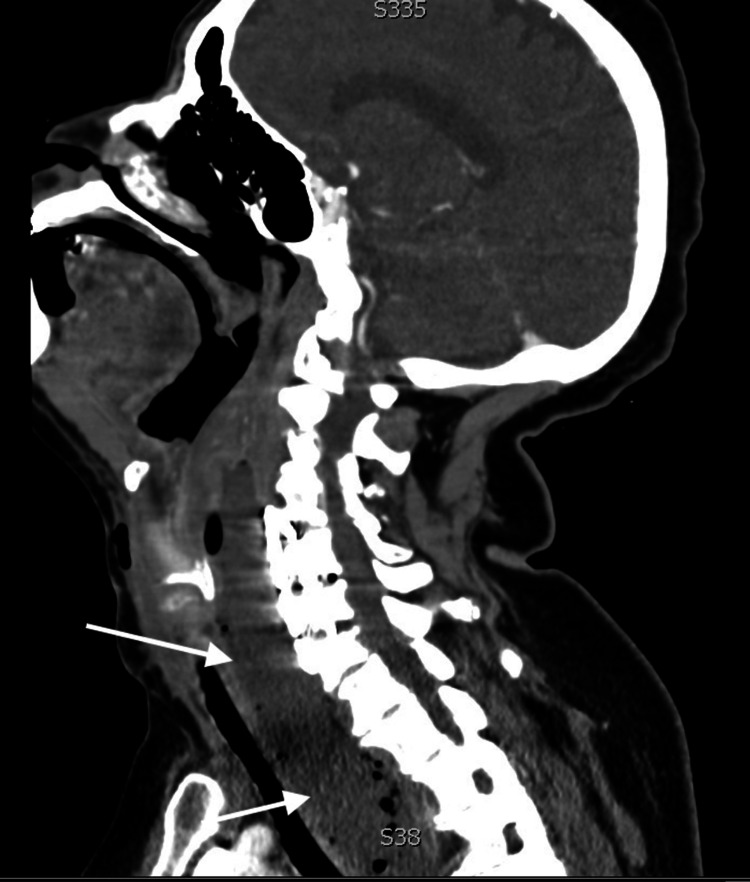
Sagittal view of a large retropharyngeal abscess with extension into the mediastinum White Arrows: Large retropharyngeal abscess with extension into the mediastinum

Contrast-enhanced computed tomography (CT) showed both a pre-platysmal abscess and a large RPAarising at the level of the cervical spine hardware with rightward mass effect on the trachea with severe glottic compression (Figure 1), subglottic narrowing (Figure 2), and mediastinal extension (Figure 3). Neurosurgery, otolaryngology, and anesthesia were consulted for definitive operative and airway management. Shortly after consultation, the patient began expelling copious purulent oral secretions. The patient's oxygen was maintained with supplemental oxygen via nasal cannula. Under local anesthetic infiltration, awake incision and drainage of both pre-platysmal and post-platysmal abscesses were performed. Approximately 30 mL of purulent fluid spontaneously drained from the abscess, after which the patient's breathing improved and the trachea returned to the midline. The incision and drainage site was left open, and rapid sequence intubation using propofol and rocuronium was performed. Following intubation, neurosurgery explored and decompressed the abscess with thorough inspection and irrigation of cervical spine hardware. Following abscess evacuation and washout, the patient was admitted to the surgical intensive care unit. Rigid esophagoscopy was performed, showing an area of purulent drainage near the esophageal inlet, suspected to be the site of perforation. Following extubation, the patient was discharged on hospital day five, vastly improved.

## Discussion

This report of a large RPA following ACDF highlights a case of life-threatening postoperative emergency airway compromise. While retropharyngeal hematoma represents the most common cause of postoperative respiratory compromise 6-24 hours following surgery, RPA becomes markedly more common between 72 and 96 hours postoperatively [1,3,4]. Airway compromise following ACDF may present insidiously over several days or rapidly over a matter of minutes [1]. Intraoperative contamination and esophageal perforation are driving etiologies for reintubation due to abscesses [1]. Patients undergoing ambulatory anterior cervical spine surgery are more likely to experience postoperative complications, with one study showing an odds ratio of 1.25 (CI, 1.06-1.49; p=.010) [5]. Postoperative RPA often presents with signs and symptoms common such as dysphagia, dysphonia, chest pain, and signs of nuchal rigidity [1,6]. Nocturnal and positional dysphagia is frequently reported, in addition to fevers and facial swelling [1,7]. 

In patients with a tenuous respiratory status, emergency physicians should consider consultation anesthesia and otolaryngology early and develop robust plans for definitive airway management assistance if decompensation occurs prior to operative management. CT imaging may demonstrate severe subglottic narrowing and glottic compression, leading to an expected difficult airway. Therefore, we believe that robust planning should include airway management considerations in the setting of glottic compression, including options such as awake intubation, mass decompression, or fiber optic intubation if the incubator is skilled and facile in such techniques. There currently exists sparse evidence regarding reintubation best practices for patients with resultant airway compromise following anterior cervical spine surgery, though rates range from 2% to 5.2% [1,8,9]. There is a lack of evidence relating to the use of supraglottic airways in the setting of failed intubation in this patient population; thus, using these tools should be at the provider's discretion. In cases where endotracheal intubation is unlikely to be successful, cricothyroidotomy is considered the intervention of choice [1,9]. In patients with airway compromise from all etiologies, rapid assessment and diagnosis are critical, as delayed airway management may lead to multiple airway attempts and resultant hypoxia, hypoxemia, and cerebral ischemia [9]. Liquids, such as sanguineous or purulent fluid, may lead to airway distortion precluding effective airway management as demonstrated in the above case [1]. As displayed in this case, incision and drainage or aspiration can be performed to restore airway anatomy prior to airway management attempts, though research supporting this practice as a routine is lacking. While there is a paucity of literature regarding this practice, this approach may be utilized in dire situations where intubation, ventilation, and cricothyroidotomy are unlikely to be successful with distorted anatomy, after definitive airway control, operative source control, and broad-spectrum antibiotics to cover the most common organisms [10]. While most retropharyngeal infections are polymicrobial, commonly identified organisms include *Staphylococcus aureus,* *Streptococcus pyogenes*, *Fusobacterium, Haemophilus* species, and anaerobic organisms [10]. Lastly, glucocorticoids may potentially be used for swelling reduction following intubation or in the setting of prolonged intubation [10].

## Conclusions

ACDF is a relatively common procedure, with numerous postoperative etiologies for airway compromise. While infrequent, postoperative airway compromise represents an anatomically challenging intubation, and it may require specialty consultation and drainage prior to establishing a definitive airway. Due to the possibility of aberrant anatomy and limited neck mobility, emergency physicians should consider deadly postoperative complications in these post-ACDF patients and prepare for a challenging airway with early specialty consultation.
